# Production of IFNβ by Conventional Dendritic Cells after Stimulation with Viral Compounds and IFNβ-Independent IFNAR1-Signaling Pathways are Associated with Aggravation of Polymicrobial Sepsis

**DOI:** 10.3390/ijms20184410

**Published:** 2019-09-07

**Authors:** Magdalena Howe, Jens Bauer, Anja Schulze, Sonja Kropp, Richard M. Locksley, Judith Alferink, Heike Weighardt, Stefanie Scheu

**Affiliations:** 1 Institute of Medical Microbiology and Hospital Hygiene, Heinrich Heine University Düsseldorf, 40225 Düsseldorf, Germany; 2 Howard Hughes Medical Institute and Departments of Medicine and Microbiology/Immunology, University of California, San Francisco, CA 94143, USA; 3 Department of Psychiatry, University of Münster, 48149 Münster, Germany; 4 Cluster of Excellence EXC 1003, Cells in Motion, 48149 Münster, Germany; 5 Life and Medical Sciences Institute (LIMES), University of Bonn, 53115 Bonn, Germany

**Keywords:** sepsis, viral infection, type I interferons, interferon β, IL-12, dendritic cells, cytokine reporter mouse model, immunotherapy

## Abstract

Viral infections are associated with increased incidence of severe sepsis. Particularly during the early stages, type I interferons (IFNs) are known mediators of detrimental effects. However, the functional role of early interferon β (IFNβ) and its cellular source during sepsis in the context of preexisting viral infections has not been defined. Using the colon ascendens stent peritonitis (CASP) model, we demonstrate that IFNβ^−/−^ and type I IFN receptor (IFNAR1)^−/−^ mice were less susceptible to sepsis after pre-stimulation with the viral mimetic poly(I:C). Wild type (WT) mice treated with poly(I:C) exhibited altered expression patterns of TNF and IL-12p40 during CASP which were dependent on IFNβ or IFNAR1, suggesting a mechanism for the increased sepsis susceptibility of WT mice. Using a double cytokine reporter mouse model, we present novel data on the simultaneous expression of IFNβ and IL-12p40 on a single cell level during polymicrobial sepsis in vivo. Conventional dendritic cells (cDCs) were identified as primary source of IFNβ and the protective cytokine IL-12p40 after CASP surgery irrespective of poly(I:C) pre-stimulation. These data demonstrated that if polymicrobial sepsis is preceded by a viral infection, IFNβ and IL-12p40 are expressed by polyfunctional cDCs suggesting that these cells can play both detrimental and beneficial roles during sepsis development.

## 1. Introduction

The millions of severe sepsis and septic shock cases reported worldwide represent a major public health threat and are associated with a mortality rate of 25% [1,2]. Severe sepsis is defined as an acute organ dysfunction caused by a dysregulated immune response after an infection [3,4,5]. During the early systemic inflammatory response excessive amounts of cytokines, e.g., type I interferons (IFNs) and tumor necrosis factor (TNF) are released by activated leukocytes and contribute to septic shock and tissue injury [6,7,8]. This is accompanied by the production of other proinflammatory cytokines, including IL-12 and IFNγ shown to have protective functions by inducing effector mechanisms essential for early bacterial clearance [9,10]. There is ample evidence that viral infections are associated with an increased susceptibility to bacterial superinfections leading to sepsis [11]. Type I IFNs are key molecules produced during such viral infections. They represent a cytokine family consisting of multiple IFNα isoforms, IFNβ, IFNω, IFNκ, and IFNε that bind to a shared heterodimeric receptor composed of two subunits (IFNAR1 and IFNAR2). Recently, type I IFNs have been implicated in the development of septic shock and associated mortality [12]. In a murine polymicrobial sepsis model, IFNAR-mediated effects were shown to be detrimental [13]. Under these conditions IFNβ is thought to be amongst the first type I IFNs to be induced and to initiate the IFNAR-mediated positive feedback loop leading to expression of other type I IFNs and IFN-induced genes. However, the specific contribution of endogenous IFNβ to the type I IFN-mediated effects remains to be determined. Furthermore, the cell type(s) responsible for type I IFN production remain undefined thereby preventing a better understanding of the cellular immune mechanisms associated with sepsis development.

This study describes the functional role of type I IFNs in early sepsis and identifies the cell type that produces IFNβ during polymicrobial sepsis for the first time in the physiological setting of a preexisting viral infection. We used the well-established bacterial sepsis model of colon ascendens stent peritonitis (CASP) and simulated viral infection by stimulation of mice with poly(I:C) before sepsis induction. Data presented in this report demonstrate that endogenous IFNβ is sufficient to dysregulate the inflammatory response elicited during the early phase of polymicrobial sepsis after pre-stimulation with poly(I:C) causing increased mortality rates. Additionally, IFNAR1-mediated signaling pathways were found to contribute to susceptibility to polymicrobial peritonitis independently of IFNβ. Using a bicistronic knock-in reporter mouse model for IFNβ and IL-12p40, we defined in a sepsis model that subpopulations of professional antigen-presenting cells, namely conventional dendritic cells (cDCs), are the major source of IFNβ and IL-12 associated with pathology and protection, respectively.

## 2. Results

### 2.1. IFNβ Deficiency Increases Early Survival in Polymicrobial Peritonitis

**Figure 1:**
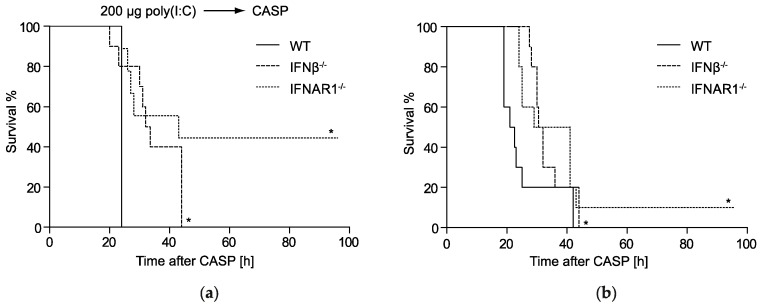
IFNβ contributes to early mortality in WT mice in polymicrobial peritonitis. Kaplan–Meier plots of survival in WT (*n* = 10), IFNβ^−/−^ (*n* = 10) and IFNAR1^−/−^ (a *n* = 9, b *n* = 10) mice pre-treated with 200 µg poly(I:C) for 24 h (**a**) or left untreated (**b**) before colon ascendens stent peritonitis (CASP). Survival was monitored for 96 h. Animals were monitored four to five times a day. * *p* < 0.05 compared to WT using log-rank test.

Virus-induced type I IFNs are essential to sensitizing the host to a secondary bacterial challenge [14,15]. However, the specific contribution of early expressed endogenous IFNβ remains undefined. To determine if IFNβ plays a functional and non-redundant role in the susceptibility to systemic bacterial infections we performed CASP surgery as an experimental sepsis model and monitored survival in IFNβ^−/−^, IFNAR1^−/−^ and WT mice. Additionally, a preceding viral infection was mimicked by poly(I:C) injection prior to CASP. We found that poly(I:C) injected IFNβ^−/−^ mice exhibited significantly longer survival times during the early phase of polymicrobial peritonitis as compared to WT mice. However, in comparison to IFNAR1^−/−^ mice survival of IFNβ^−/−^ mice was reduced (Figure 1a). Without poly(I:C) challenge WT mice were significantly more susceptible to CASP than IFNAR1^−/−^ mice, in accordance with earlier findings [13]. Also, in this model, IFNβ^−/−^ mice exhibited a significantly increased survival in comparison to WT mice (Figure 1b).

These data suggest a non-redundant detrimental role of endogenously produced IFNβ during the early phase of polymicrobial peritonitis.

### 2.2. IFNAR1 Deficiency, but Not IFNβ Deficiency Prevents an Increase in Bacterial Counts Early after Poly(I:C) Sensitization

**Figure 2:**
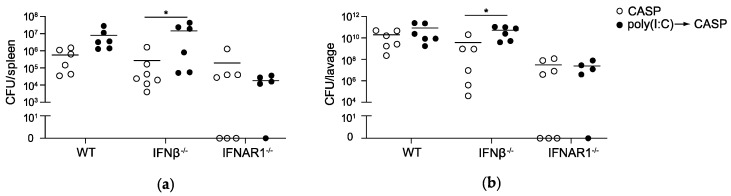
Increase in the bacterial load early during CASP after poly(I:C) pre-treatment in IFNβ deficiency but not IFNAR1 deficiency. WT, IFNβ^−/−^ and IFNAR1^−/−^ mice were injected with 200 µg poly(I:C) followed by CASP surgery. Bacterial load in the spleen (**a**) and peritoneal lavage (**b**) was determined 12 h after CASP; *n* = 5–7 animals per group. * *p* < 0.05 using Student’s *t*-test.

To elucidate the mechanisms underlying the reduction in mortality rates during polymicrobial sepsis in IFNβ^−/−^ and IFNAR1^−/−^ mice in the presence vs. absence of a poly(I:C) pre-stimulation, we analyzed the antibacterial host response in the mouse strains. Bacterial loads in the important exemplary organs spleen and peritoneal cavity after CASP combined with poly(I:C) pre-treatment were compared to bacterial loads during CASP alone in WT, IFNβ^−/−^ and IFNAR1^−/−^ mice (Figure 2).

In IFNβ^−/−^ and WT mice, CFUs 12h after CASP were comparable in the absence of poly(I:C) pre-treatment. However, following poly(I:C) pre-stimulation, bacterial loads in the spleen (Figure 2a) and peritoneal lavage fluid (Figure 2b) were slightly increased as compared to CASP alone in WT and IFNβ^−/−^ mice, with differences reaching significant levels only in IFNβ^−/−^ mice. In contrast, poly(I:C) treatment did not affect bacterial numbers in IFNAR1^−/−^ mice exposed to CASP. At 24h after CASP no significant differences in CFU counts in the spleen and peritoneal lavage were detectable between poly(I:C) treatment and no pretreatment in either genotype (Figure S1a,b). These data indicate that until 12 h after CASP, deficiency in IFNAR1, but not IFNβ alone, is sufficient to prevent an increase in bacterial counts in lymphoid organs such as the spleen and body fluids such as peritoneal fluid after poly(I:C) treatment. Thus, a lower mortality of IFNβ^−/−^ mice early after CASP is observed despite an increase in the bacterial load in the spleen and the peritoneal cavity.

### 2.3. Dysregulated IL-12p40 Production during Septic Peritonitis Following Poly(I:C) Treatment

While our results indicate that IFNβ contributes to increased mortality under septic conditions independent of poly(I:C) pre-treatment, production of the proinflammatory cytokine IL-12p40 has been shown before to be protective in humans as well as in animal models [10,16]. We therefore elucidated the specific role of IFNβ in the modulation of the IL-12 response in the CASP model.

**Figure 3:**
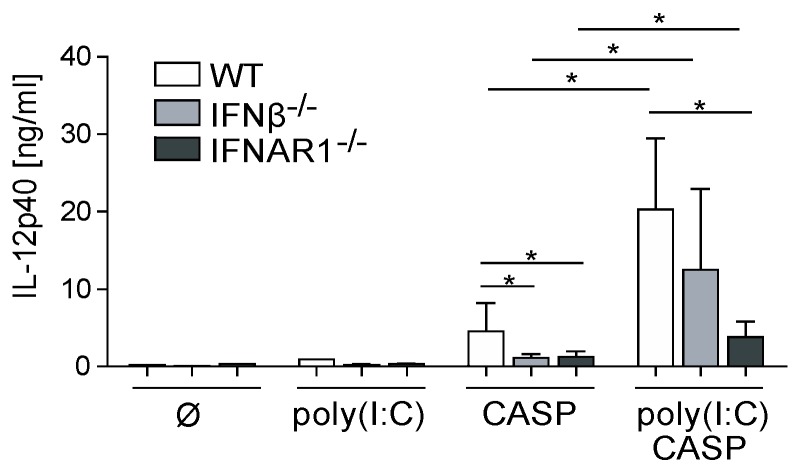
Differential effects of poly(I:C) pre-treatment on cytokine production during CASP. WT, IFNβ^−/−^ and IFNAR1^−/−^ mice were pre-treated with 200 µg poly(I:C) followed by CASP surgery. IL-12p40 concentrations in the serum were determined by ELISA. *n* = 5–7 animals per group. Error bars indicate SD. * *p* < 0.05 using Student’s *t*-test.

After CASP, we found equally reduced systemic IL-12p40 levels in IFNβ^−/−^ and IFNAR1^−/−^ mice as compared to WT mice (Figure 3). Poly(I:C) administration increased IL-12p40 levels in WT, IFNβ^−/−^ and IFNAR1^−/−^ mice subjected to CASP within the respective genotype as compared to animals after CASP alone. However, after poly(I:C) pre-treatment IFNAR1^−/−^ but not IFNβ^−/−^ mice exhibited significantly lower systemic IL-12p40 levels as compared to WT animals. These data indicate that prestimulation with viral compounds enhances the protective IL-12 response during polymicrobial peritonitis in the absence of IFNβ.

### 2.4. Conventional DCs Represent the Primary Source of IFNβ and IL-12p40 After CASP

The cellular sources of type I IFNs or IL-12 production during sepsis remain unknown. To visualize IFNβ vs. IL-12p40 production on a single cell basis in vivo, we crossed previously generated IFNβ/YFP knock-in reporter mice (IFNβ^mob/mob^) [17] with a reporter mouse model for IL-12p40/GFP (IL-12p40^get40/get40^) [18] and generated the double reporter mouse line (IFNβ^mob/mob^ × IL-12p40^get40/get40^). Myeloid cell populations encompassing inflammatory monocytes, various dendritic cell subsets, macrophages, and neutrophils represent the most important cellular candidates for IFNβ and IL-12 production in the spleen. We and others have demonstrated before, that defining a reliable gating strategy for detection of IFNβ producing cells ex vivo is challenging due to the presence of limited numbers of these cells in vivo [17,19,20,21]. Our pilot studies indicated that also in this sepsis model low numbers of IFNβ/YFP-positive and IFNβ/YFP × IL-12p40/GFP-double positive cells are present in the spleen of the respective reporter mice (data not shown). We therefore used the reference model of *Listeria monocytogenes* infection, where we and others have defined before reliable gating strategies for detection of low numbers of IFNβ producing myeloid cells in the spleen of IFNβ^mob/mob^ mice [20,22,23]. In this reference model the gating strategy also in IFNβ^mob/mob^ × IL-12p40^get40/get40^ mice for detection of myeloid cells that produce IFNβ/YFP alone or co-express IL-12p40/GFP was now defined. At 24h after infection with *L. monocytogenes*, myeloid cells in the spleen can be separated into CD11c^low^ CD11b^+^ cells (Figure S2a, gate G1) and CD11c^hi^ cDCs (Figure S2a, gate G2). CD11c^low^ CD11b^+^ cells (gate G1) are a heterogeneous subpopulation consisting of CD11b^+^ Ly6C^low^ Ly6G^low^ non-inflammatory myeloid cells, CD11b^+^ Ly6C^hi^ Ly6G^low^ cells characterized before as inflammatory monocytes, and CD11b^+^ Ly6C^int^ Ly6G^hi^ neutrophils (Figure S2a). In accordance to earlier findings, in naïve IFNβ^mob/mob^ × IL-12p40^get40/get40^ mice, the main producers of IL-12p40/GFP were identified as CD11c^hi^ cDCs, the majority of which express CD8α (Figure S2b) [18].

**Figure 4:**
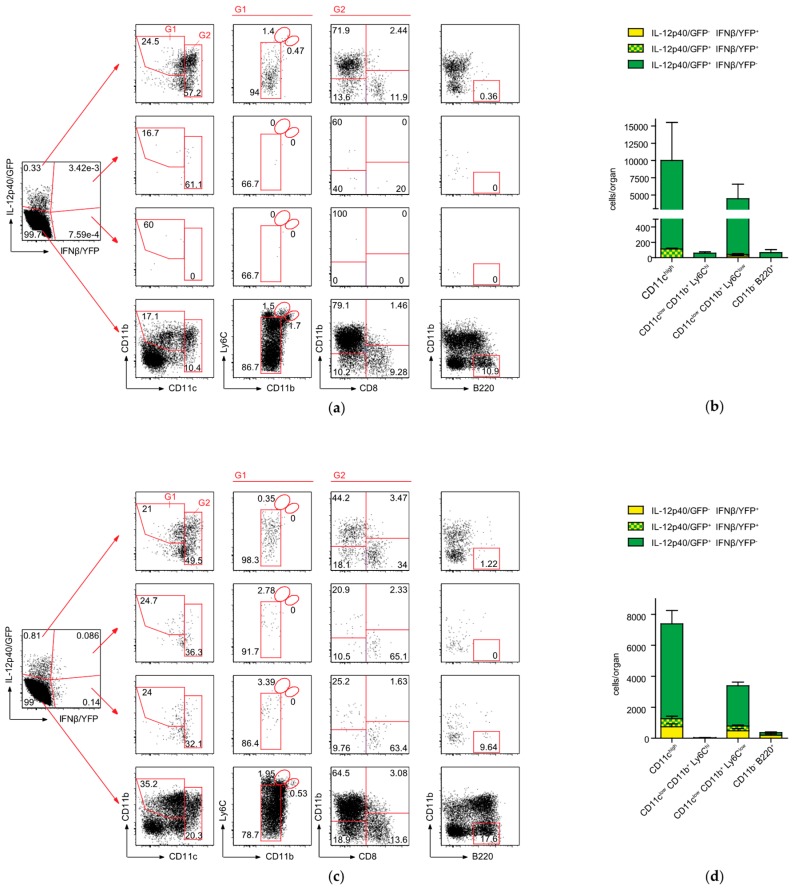
Conventional dendritic cells (DCs) are the main producers of IFNβ and IL-12p40 after CASP. IFNβ^mob/mob^ × IL-12p40^get40/get40^ mice were left untreated or stimulated with 200 µg poly(I:C) for 24 h followed by CASP. At 16 h after CASP, spleen cells were analyzed by flow cytometry for IFNβ/YFP and IL-12p40/GFP expression. Phenotypic analysis of IFNβ/YFP and IL-12p40/GFP expressing cells after (**a**) CASP or (**c**) after poly(I:C) stimulation followed by CASP in IFNβ^mob/mob^ × IL-12p40^get40/get40^ mice. The cell populations were electronically pre-gated on CD19^−^ CD3ε^−^ live cells. Total cell numbers in the spleen were calculated for cells expressing IFNβ/YFP and/or IL-12p40/GFP gated on CD11c^high^ (cDCs), CD11c^low^ CD11b^+^ Ly6C^hi^ (inflammatory monocytes), CD11c^low^ CD11b^+^ Ly6C^low^ (non-inflammatory myeloid cells, and CD11b^−^ B220^+^ (pDCs) as indicated after (**b**) CASP or (**d**) after poly(I:C) stimulation for 24 h followed by CASP in IFNβ^mob/mob^ × IL-12p40^get40/get40^. *n* = 3 animals per group. Error bars indicate SEM. Shown is one representative experiment of five independent experiments.

During CASP without poly(I:C) pre-treatment, the majority of cells expressing either IL-12p40 or both IFNβ and IL-12p40 were defined as CD11c^hi^ cDCs (gate G2; Figure 4a). Lower frequencies of CD11c^low^ CD11b^+^ Ly6C^low^ non-inflammatory myeloid cells and an even smaller fraction of B220^+^ plasmacytoid DCs (pDCs) were also found to express IL-12p40/GFP after CASP surgery (Figure 4a,b). In contrast to the minimal but still detectable frequencies of low IFNβ/YFP expressing cells in the spleen after CASP, significantly elevated frequencies and total numbers of IFNβ-expressing (*p* = 0.03) but not IFNβ/IL-12p40- (*p* = 0.06) and IL-12p40-expressing cells (*p* = 0.23) were detected following CASP after poly(I:C) pre-treatment in cDCs, pDCs, or CD11c^low^ CD11b^+^ Ly6C^low^ non-inflammatory myeloid cells (Figure 4c,d). Here, the majority of IL-12p40 and IFNβ single or coproducing cells were cDCs and in all three subpopulations of cDCs analyzed, CD11b^−^ CD8α^−^, CD11b^+^ CD8α^−^, as well as CD11b^−^ CD8α^+^, IL-12p40/GFP and/or IFNβ/YFP expression was detectable (Figure 4c). Additionally, under these conditions around 3% of IFNβ-expressing as well as IL-12p40/IFNβ-coexpressing CD11c^low^ CD11b^+^ myeloid cells were identified as Ly6C^hi^ inflammatory monocytes (Figure 4c).

Approximately half of all IFNβ-expressing cells also produced IL-12p40 during peritoneal sepsis independent of a poly(I:C) treatment. No IFNβ/YFP or IL-12p40/GFP expression was detectable in WT control mice after CASP with or without poly(I:C) pre-treatment (Figure S2c). In sum, these data demonstrate that during sepsis the functionally opposed cytokines IFNβ and IL-12p40 are expressed primarily by a population of polyfunctional cDCs and CD11b^+^ myeloid cells.

### 2.5. TNF and IL-10 Levels in the Peritoneal Lavage during Septic Peritonitis Are Differentially Modulated Following Poly(I:C) Challenge

**Figure 5:**
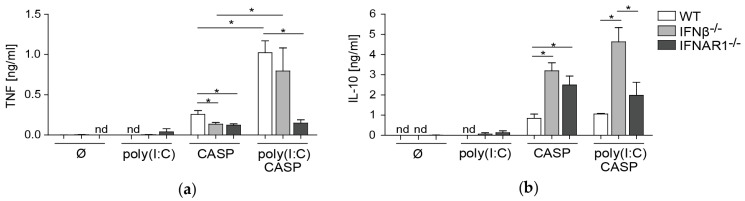
Differential effects of poly(I:C) pre-treatment on pro- vs. anti-inflammatory cytokine levels in the peritoneal cavity during CASP. Mice were treated as described in Figure 2 and Figure 3. Peritoneal fluid was harvested before CASP (∅) or 12 h thereafter. TNF (**a**) and IL-10 (**b**) concentrations in the peritoneal fluid were determined by ELISA. *n* = 5–7 animals per group. Error bars indicate SD. * *p* < 0.05 using Student’s *t*-test.

Polymicrobial peritonitis is accompanied by a dysregulated cytokine response, mediated in large part by TNF [24,25]. Following CASP, it was shown that expression of proinflammatory cytokines like TNF were positively regulated by type I IFNs based on the observation that IFNAR1^−/−^ mice exhibited lower levels of these factors [12,13]. We thus investigated levels of this innate pro-inflammatory cytokine in the peritoneal lavage in our comparative sepsis model. TNF levels in the peritoneal fluid were reduced after CASP in IFNβ^−/−^ and IFNAR1^−/−^ mice as compared to WT mice (Figure 5a). However, after poly(I:C) pre-treatment, TNF levels were markedly increased in WT and IFNβ^−/−^ but not in IFNAR1^−/−^ mice. In addition, under these conditions, TNF levels were lower in IFNAR1^−/−^ mice as compared to WT mice. This is indicative of a highly proinflammatory environment generated by IFNAR-mediated signaling events that in the absence of early production of IFNβ can be compensated by other type I IFN subtypes. We further investigated levels of the anti-inflammatory cytokine IL-10 in the peritoneal lavage in this model. After CASP, IL-10 levels were markedly enhanced in IFNβ^−/−^ and IFNAR1^−/−^ mice as compared to WT mice (Figure 5b). Interestingly, after prior poly(I:C) treatment, IFNβ^−/−^ mice exhibited higher systemic levels of IL-10 as compared to WT and IFNAR1^−/−^ mice after CASP surgery. In the complete absence of IFNAR-mediated signaling in IFNAR1^−/−^ mice under these conditions, increases in IL-10 production were less pronounced. These data suggest that IFNβ also modulates production of IL-10 in the peritoneal cavity during CASP following a viral challenge.

This side-by-side analysis of bacterial peritonitis in the presence or absence of a viral pre-stimulation in IFNβ^−/−^ versus IFNAR1^−/−^ mice revealed a previously unknown function of IFNβ in the regulation of pro- and anti-inflammatory cytokine production during early sepsis development.

## 3. Discussion

The finding that primary viral infections predispose patients to severe sepsis has been confirmed by the observation that outbreaks of e.g., influenza often go along with an increase in frequencies of reported sepsis cases [11,26,27]. In general, bacterial superinfections during an ongoing viral infection are associated with more severe and prolonged symptoms. Also, in several murine infection models non-lethal bacterial challenges progress to lethal infections, if viral stimulation precedes the bacterial infection. Recent data identified the inflammasome as another key player in a model of acute septic shock and poly(I:C) stimulation prior to exposure to LPS was shown to be detrimental due to caspase-11 activation [28,29]. Experimental models of co-infections are therefore highly relevant to the development of novel therapeutic strategies. Using a model of polymicrobial sepsis combined with prior viral stimulation this study revealed novel functions of IFNβ that critically impact the outcome of sepsis. Earlier studies demonstrated that type I IFNs are involved in lethality following septic shock and the murine CASP model of polymicrobial sepsis model [12,13,30]. In our study direct comparisons between WT, IFNβ^−/−^ and IFNAR1^−/−^ mice indicated that poly(I:C)-induced IFNβ production has a non-redundant, detrimental role in IFNAR-mediated sensitization leading to increased mortality rates early during polymicrobial sepsis. The selective function of IFNβ in our co-infection model is supported by data on its ability to bind to IFNAR1 in an IFNAR2 independent manner activating a unique set of genes in LPS shock [31]. The fact that survival of IFNAR1^−/−^ mice was improved after poly(I:C) pre-treatment indicates that in the absence of IFNAR1, poly(I:C) activates protective immune functions in this peritoneal sepsis model if undisturbed by a type I IFN response. Regarding the sensory pathway that is activated by poly(I:C) treatment *in vivo*, Gitlin et al. and McCartney et al. have shown that poly(I:C) mediated IFNβ induction is mediated via the MDA5 pathway, while IL-12p40 expression is dependent on TLR3 [32,33]. Additional studies are needed to define the protective mechanisms elicited by poly(I:C) stimulation followed by CASP in IFNAR1^−/−^ mice.

A dysbalanced immune activation leads to immunopathologies associated with detrimental outcomes. During the course of polymicrobial sepsis excessive production of proinflammatory cytokines, e.g., TNF or IL-1β, is dependent on IFNAR-mediated signaling [12]. In our studies a poly(I:C)-induced increase in TNF levels was dependent on IFNAR1 but not on IFNβ expression, while IL-10 production, known to mediate protective functions in the CASP model [34], was higher in IFNβ^−/−^ mice compared to WT and IFNAR1^−/−^ mice. This suggests that this immunosuppressive cytokine might counterbalance the proinflammatory immune dysregulation observed during the early stages of sepsis [35]. Therefore, the elevated levels of IL-10 observed in the absence of IFNβ likely contribute to the reduction in mortality in IFNβ mice compared to WT mice after CASP independent of poly(I:C) pre-treatment. This, however, needs to be functionally proven by IL-10 blocking experiments or by using IFNβ^−/−^ IL-10^−/−^ double deficient mice in future studies. In IFNAR1^−/−^ mice the poly(I:C)-induced dysregulation was not as pronounced as in IFNβ^−/−^ mice suggesting that the immunosuppressive feedback regulation likely mediated by IL-10 was less critical and not activated to the same degree in IFNAR1^−/−^ mice compared to IFNβ^−/−^ mice. However, sustained elevated levels of IL-10 may be detrimental during the late phase of sepsis due to its immunosuppressive properties that may negatively impact bacterial clearance [35]. Data presented in this report demonstrate that IFNβ significantly impacts progression of the early phase of polymicrobial peritonitis by contributing to the dysregulated production of pro- and anti-inflammatory cytokines. It thereby affects the delicate balance between protective and detrimental host responses that determine the outcome of polymicrobial peritonitis.

Due to the lack of sensitive analytic tools the identity of the immune effector cells responsible for the production of IFNβ and IL-12p40 during CASP remained unknown. In our earlier studies, utilizing the IFNβ^mob/mob^ model, we identified IFNβ-expressing cells after in vivo poly(I:C) administration predominantly as cDCs [17]. We now demonstrate that irrespective of a poly(I:C) pre-stimulation the majority of IFNβ and IL-12p40 producing cells in the CASP sepsis model were CD11c expressing cDCs. When comparing the frequencies of cytokine-producing cells to the serum level of the cytokine it became apparent that poly(I:C) stimulation prior to polymicrobial peritonitis leads to increased levels of IL-12p40 per cell rather than an increase in the number of cells producing this cytokine. Conversely, a significant increase in the number of single IFNβ and IFNβ-IL-12p40-coproducing cells was observed following poly(I:C) pre-treatment. However, in both cases a limited number of cells were responsible for the production of biologically significant amounts of IL-12p40 or IFNβ, the hallmark cytokines for a protective versus a detrimental immune response during sepsis. The low frequency of type I IFN producing cells after bacterial and viral stimulation is supported by previous reports using the IFNβ^mob/mob^ model as well as other independently generated IFNβ and IFNα6 reporter mice [17,20,21,36]. At the same time, the low numbers of type I IFN producing cells detected here in the IFNβ^mob/mob^ reporter mice at the single cell level hampered further phenotypic and functional analysis on this rare cell population. Future studies comprising e.g., single cell transcriptomics, in vivo ablation of CD11c+ cells, and mice with a cell type specific deficiency in IFNβ promise to shed light on these important immune effector cells. Also, the specific and possibly even opposing roles of the IL-12p40 subunit in polymicrobial sepsis as part of the IL-12p70 or IL-23 heterodimers or as IL-12p40 homodimers await clarification.

In conclusion, we demonstrate in a clinically relevant model of polymicrobial sepsis in the context of a preceding viral stimulation that IFNβ represents an early critical mediator of the aggravation of sepsis in the early phase of disease. Presence of IFNβ was associated with higher mortality and a dysregulated cytokine profile. A small subset of cDCs was identified as the cellular source of the detrimental IFNβ as well as the protective IL-12p40. The identification of these cytokine producing effector cells contributes further to a better understanding of host cellular response mechanisms during sepsis. At the same time, our data indicate an IFNβ-independent detrimental role of IFNAR1-mediated signaling pathways in polymicrobial peritonitis. In addition, this work opens the door for the development of novel therapeutic interventions targeting cytokine expression specifically in DCs that can mediate the elicitation of protective immune response to secondary bacterial infections.

## 4. Materials and Methods

### 4.1. Mice, Colon Ascendens Stent Peritonitis (CASP), Poly(I:C) Stimulation, and Listeria Monocytogenes Infection

Female C57BL/6 mice were purchased from Charles River. Female mice were chosen due to the lower variance in body weight between individuals as compared to male mice. IFNβ^mob/mob^ (*m*essenger *o*f IFN *b*eta: IFNβ/YFP reporter mouse) [17], IL-12p40^get40/get40^ mice (*g*reen *e*nhanced *t*ranscript for *p40*: IL-12p40/GFP reporter mouse) [18], IFNβ^−/−^ [37], and IFNAR1^−/−^ [38] mice were backcrossed for at least 10 generations onto C57BL/6 background and housed under specific pathogen-free conditions in the animal research facility of the University of Duesseldorf. Mice at 8–12 weeks of age were used for all experiments. Mice were injected intravenously with 200 µg poly(I:C) (GE Healthcare, Freiburg, Germany) 24 h before CASP surgery. Polymicrobial sepsis was induced through CASP surgery as described in detail previously [9]. Mice were anesthetized with Ketamine (100 mg/kg) and Xylazine (5 mg/kg). The colon ascendens was exteriorized and a 7/0 nylon non-absorbable thread (Resorba, Nürnberg, Germany) was stitched through the antimesenteric wall into the lumen of the colon ascendens approximately 10 mm distal to the ileocecal valve. A 14 gauge venous catheter was punctured through the colonic wall into the intestinal lumen and fixed. Feces were massaged from the cecum into the colon ascendens and the stent was fixed. Fluid compensation was performed by flushing 0.5 ml of sterile saline into the peritoneal cavity followed by closure of the abdominal wall. For *Listeria* infection 10^7^ CFU *L. monocytogenes* (EGD) were injected into the peritoneal cavity as described previously [20]. Spleens were harvested 24 h after infection and analyzed by flow cytometry. All animal experiments were approved by the government of North-Rhine Westphalia.

### 4.2. Bacterial Counts

Mice were sacrificed either before or 12 h after CASP surgery. The spleen was isolated and homogenized and peritoneal lavage fluid was collected. Serial dilutions were performed in PBS and plated on sheep blood agar plates (Biomerieux, Nürtingen, Germany). Colonies were counted after 24 h incubation at 37 °C and CFUs were calculated per whole spleen or peritoneal cavity.

### 4.3. Cytokine Production

Peritoneal lavage fluid and peripheral blood were collected before and 12 h after CASP surgery. Cytokine concentrations were determined in serum and peritoneal lavage fluid by ELISA specific for IFNβ (PBL, Piscataway, NJ, USA), IL-12p40 (BD Biosciences, Heidelberg, Germany), TNF and IL-10 (R&D Systems, Mainz, Germany) according to manufacturer’s instructions.

### 4.4. Antibodies

We used CD3ε (145-2C11), CD19 (1D3), B220 (Ra3-6B2), CD11c (HL3), CD11b (M1/70), Ly6C (AL-21), CD8α (53–6.7) from BD Biosciences, Ly6G (1A8) from BioLegend (London, UK) and CD16/CD32 (clone 93) from eBioscience (San Diego, CA, USA).

### 4.5. FACS Analysis

The isolated spleens were digested with collagenase VIII (Sigma, Taufkirchen, Germany) and DNase I (Roche, Mannheim, Germany) and stained in parallel. Coexpression of indicated cell surface markers with YFP and GFP expression was analyzed on a FACS Canto II (BD Biosciences, Heidelberg, Germany). IFNβ/YFP and IL-12p40/GFP gating was adjusted to equally treated WT, IFNβ^mob/mob^ and IL-12p40^get40/get40^ mice.

### 4.6. Statistical Analysis

Statistical analysis for bacteria and cell counts and cytokine measurement was performed using the Student’s *t*-test. For survival log-rank test was used. Data are represented as means. Error bars represent SD or SEM as indicted.

## Supplementary Materials

Supplementary Materials can be found at https://www.mdpi.com/1422-0067/20/18/4410/s1.

## Abbreviations

CASP	Colon Ascendens Stent Peritonitis
cDC	conventional Dendritic Cell
CFU	Colony Forming Unit
DC	Dendritic Cell
ELISA	Enzyme Linked Immunosorbent Assay
GFP	Green Fluorescent Protein
IFN	Interferon
IFNAR	Type I IFN Receptor
IL	Interleukin
LPS	Lipopolysaccharide
pDC	plasmacytoid Dendritic Cell
poly(I:C)	polyinosinic polycytidylic acid
SD	Standard Deviation
SEM	Standard Error of Mean
TNF	Tumor Necrosis Factor
WT	Wildtype
YFP	Yellow Fluorescent Protein
