# Supplementary material for: Production of IFNβ by Conventional Dendritic Cells after Stimulation with Viral Compounds and IFNβ-Independent IFNAR1-Signaling Pathways are Associated with Aggravation of Polymicrobial Sepsis
Source: Int J Mol Sci. 2019 Sep 7;20(18):4410. doi: 10.3390/ijms20184410 (PMC6770674; doi:10.3390/ijms20184410)
Supplement: Supplementary file 1 [file ijms-20-04410-s001.pdf]

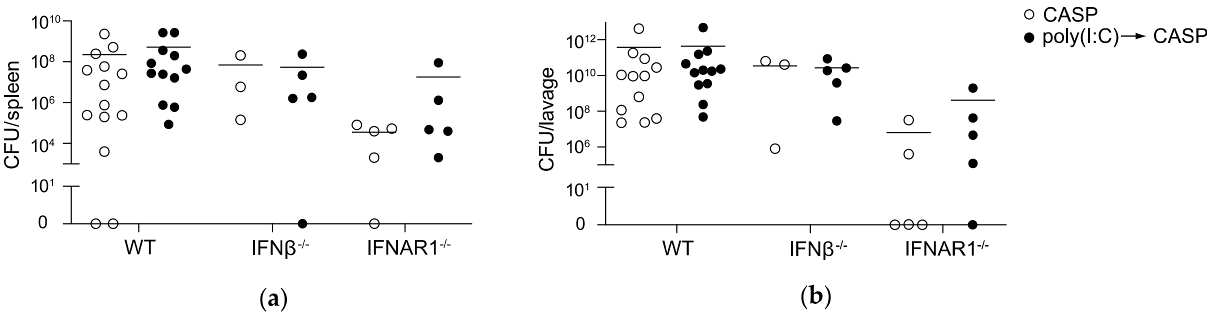

Figure S1. Unaltered bacterial loads 24h after CASP after poly(I:C) pre-treatment. WT, IFNβ$^{-/-}$ and IFNAR1$^{-/-}$ mice were injected with 200 µg poly(I:C) followed by CASP surgery. Bacterial load in the spleen (**a**) and peritoneal lavage (**b**) was determined 24h after CASP; n = 3–12 animals per group. * $p < 0.05$ using Students t-test.

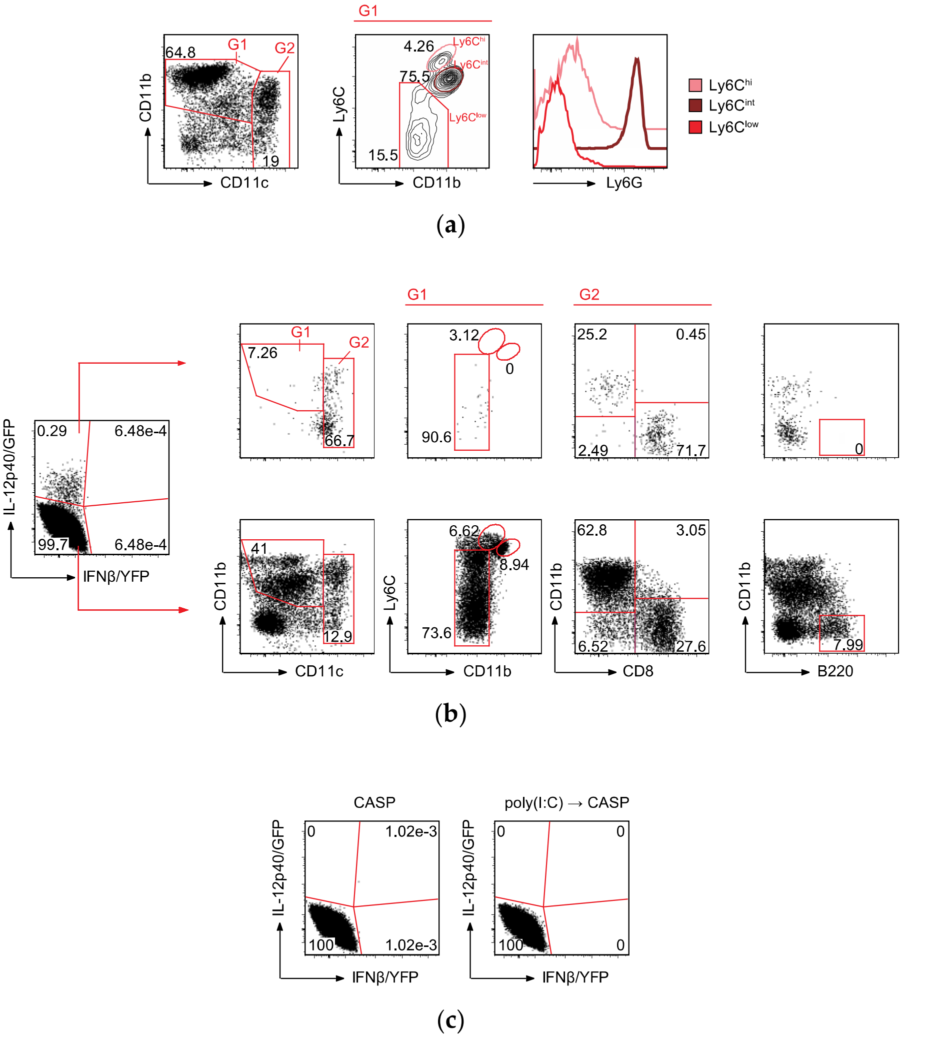

Figure S2. Gating strategy for the definition of IFNβ and/or IL-12p40 producing cells in the spleen. The cell populations were electronically gated on CD19$^-$, CD3ε$^-$ live cells. (**a**) WT mice were infected intraperitoneally with 10^7 CFU *L. monocytogenes* and spleens analyzed after 24 h by flow cytometry. (**b**) IFNβ$^{mob/mob}$ x IL-12p40$^{get40/get40}$ mice were left untreated. Cells were phenotypically analyzed by flow cytometry for IFNβ/YFP and IL-12p40/GFP expression. (**c**) Flow cytometric analysis of YFP and GFP fluorescence in the spleen of WT mice after CASP with or without poly(I:C) pretreatment. n = 2–11. Shown is one representative experiment of two to three independent experiments.
